# Indole Alkaloids as Biased Opioid Receptor Modulators

**DOI:** 10.3390/ph19030397

**Published:** 2026-02-28

**Authors:** Oliver Grundmann, Allison Henderson

**Affiliations:** Department of Medicinal Chemistry, College of Pharmacy, University of Florida, Gainesville, FL 32611, USA; henderson.a@ufl.edu

**Keywords:** indole, opioid, beta-arrestin, natural products, respiratory depression

## Abstract

**Background**: Opioid receptors are a commonly used target for treatment of pain conditions. Most opioids used in therapy are linked to adverse effects such as tolerance, dependence, and respiratory depression. Indole alkaloids acting on opioid receptors may provide a novel molecular mechanism to confer analgesic effects. **Results**: Indole alkaloids such as ibogaine and mitragynine act on μ-opioid receptors as biased full or partial agonists that do not, or much less strongly, recruit β-arrestin compared to non-biased agonists. The recruitment of β-arrestin has been linked to adverse effects, most notably substantial respiratory depression. The molecular mechanism of biased activation has been proposed to be associated with accommodation of the indole structure that leads to a different spatial orientation of amino acid residues in transmembrane regions 2 and 3 of the μ-opioid receptor as well as extracellular helix 8. **Conclusions**: Naturally occurring indole alkaloids show biased G-protein coupled activation of opioid receptors with limited recruitment of β-arrestin, thus limiting commonly observed adverse effects. Indole alkaloids may present a feasible structure to develop new biased opioid modulators with an improved risk-to-benefit ratio.

## 1. Introduction

Opioid receptors remain an essential target for drugs to treat acute and chronic pain disorders. Ever since the discovery and isolation of morphine from the unripe seed pods of the poppy plant (*Papaver somniferum*, Papaveraceae), semi-synthetic and synthetic substances have been entering the market. Many of them have substantially impacted the treatment approach for pain disorders and have advanced our understanding of opioid receptor modulation. The discovery of the G-protein-coupled opioid receptors subsequently led to the identification of endogenous peptides that bind and activate the second messenger cascade to relieve pain. Dynorphins, enkephalins, and nociceptin have been identified as endogenous opioid receptor agonists with varying affinity at μ-, κ-, δ-, and nociception opioid receptors [1,2,3]. Despite the historical advances that have been made in elucidating the specific function of opioid receptors in pain relief, it is well-established that each opioid receptor also leads to adverse effects that are dose-limiting and may lead to risk of dependence [4].

All opioid receptors are inhibitory G-protein coupled receptors. While μ- and κ-opioid receptors are the primary targets of currently marketed drugs, the more recently discovered nociceptin receptor has become a desirable target for new treatment options for anxiety, depression, and eating disorders. However, many current drugs that act on nociceptin receptors also activate the other opioid receptors, thus contributing to both desirable and undesirable effects.

Activation of opioid receptors impacts a range of physiological processes. Depending on the location of the receptor and the specific receptor activated, the response can range from therapeutic pain relief to potential fatal respiratory depression. Activation of δ-opioid receptors in the central nervous system causes analgesia, convulsions, and physical dependence, as well as modulate μ-opioid receptor induced respiratory depression [5]. Despite the involvement of δ-opioid receptors in some of the effects of approved opioid medications, there are currently no δ-opioid receptor selective drugs on the market [6]. While κ-opioid receptor activation also causes analgesia, other effects include anticonvulsant and depressive symptoms as well as dysphoria, diuresis, sedation, and miosis. It does also relieve stress, although this effect may be indirectly mediated through inhibition of cortisol release [7,8]. The classical symptoms of opioid toxicity are mediated through activation of the three μ-opioid receptor subtypes, with μ1 receptors leading to physical dependence and analgesia, and μ2 receptors causing respiratory depression, miosis, euphoria, constipation, and physical dependence. While μ3 receptors are not well-researched, activation may lead to peripheral and central vasodilation [9].

Given the widespread use of medications that target the opioid receptors for treatment of acute and chronic pain conditions, the adverse effects of physical dependence and respiratory depression remain of great concern [10]. Between 1999 and 2024, a total of 806,765 individuals in the US have died from an opioid-involved overdose with the predominant cause being respiratory depression [11,12]. Medications that activate opioid receptors have tremendous therapeutic value and are among the most prescribed medications for analgesia. However, research has been ongoing to identify more selective opioid receptor agonists absent or with substantially reduced respiratory depression liability.

## 2. Indole Alkaloid Opioid Receptor Modulators and Agonists

Since the discovery of opium and isolation of morphine by the German pharmacist Friedrich Sertürner in 1804 [13], the elucidation of morphine’s structure led to the initial testing of pharmacodynamic structure–activity principles for agonists at opioid receptors. In 1976, Feinberg et al. proposed essential structural features for opioid agonists: a lipophilic binding site, an amine binding site, and an agonist binding pocket. In addition, for mixed or full opioid antagonists, an additional binding interaction had to occur [13,14]. While almost all opioids do meet these structural features, there have been deviations such as the κ-opioid receptor agonist salvinorin A that is a neoclerodane diterpene lacking a nitrogen [15,16].

Morphine as the prototypical opioid interacts with the murine μ-opioid receptor through the following amino acid residues: ASP147, MET151, TRP293, ILE296, HIS297, VAL300, ILE322, and TYR326 [17,18]. Although not clearly established, additional interactions with residues ASN150 and VAL236 may lead to antagonist activity, as is reported for naloxone. The benzomorphinan backbone of morphine and structurally related substances confer substantial rigidity on the molecule, which may limit the potency of such compounds and lead to an unbiased activation of opioid receptors.

**Figure 1:**
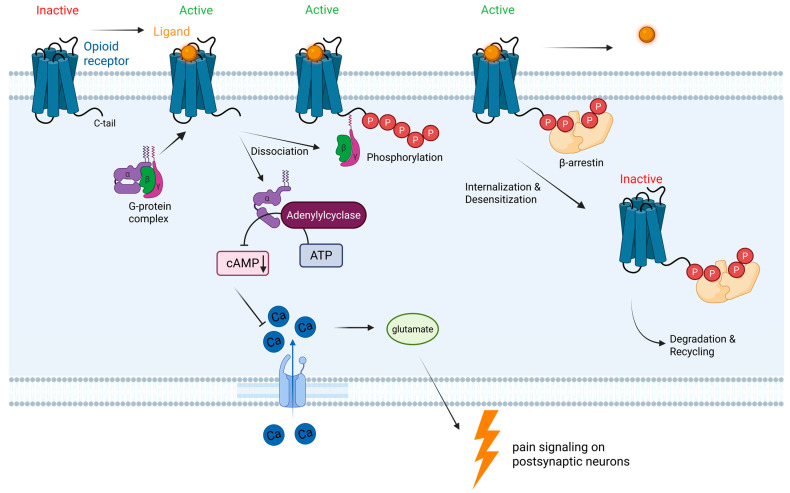
Illustration of opioid G-protein coupled receptors and second messenger pathways leading to differential pharmacological effects through activation of the G protein complex and β-arrestin. Created in BioRender. Grundmann, O. (2026). https://BioRender.com/8jmd15u (accessed on 25 February 2026).

G-protein coupled receptors are associated with the concept of biased activation, with two intracellular pathways being activated [19]. The decreased generation of the secondary messenger cAMP is associated with pain relief through decreased calcium influx and subsequent release of glutamate that would activate the post-synaptic neuron to signal pain (Figure 1). The recruitment of the β-arrestin following phosphorylation of the still active opioid receptor signals receptor internalization. This leads to a reduction in available opioid receptors on the cell surface, and hence, desensitization. Repeated use of opioids reduces the analgesic’s effectiveness and, through the β-arrestin pathway, leads to associated adverse effects such as respiratory depression, constipation, dependence, and tolerance [20]. While opioids like morphine do activate both the intracellular second messenger cascade for GPCRs and β-arrestin, a number of biased ligands have a preference for activating one pathway over another. In particular, activation of the β-arrestin pathway in opioid receptors has been associated with tolerance development to opioids and desensitization of receptors to ligand binding (Figure 1). For μ-opioid receptors, activation of β-arrestin 2 appears to be associated with slowed gastrointestinal motility, reduced respiratory rate, and development of tolerance to opioids [21,22,23]. This has led to the search for biased GPCRs at opioid receptors that do not, or to a much lesser degree, activate β-arrestin 2 as a new treatment modality for pain with potentially less adverse effects, especially respiratory depression.

A recent structural investigation identified a specific conformational change in the μ-opioid receptor that specifically activates the β-arrestin pathway. The amino acid residues are located in transmembrane region 1 of the μ-opioid receptor that is linked to an intracelullar loop helix-8 [24]. Of substantial contribution to the activation of β-arrestin is an arginine residue in location 86 in transmembrane 1, which mediates recruitment in conjunction with a tyrosine residue in position 75. Mutagenic replacement of arginine confirmed that β-arrestin recruitment is substantially impeded, while G-protein recruitment remained at 40–60% for inhibitory activation of second messenger cascades, the primary activity for analgesia.

An important note relates to the ongoing discussion of the separation of G-protein-coupled activation from the β-arrestin pathway. While some research indicates that biased agonism of GPCRs in the absence of β-arrestin recruitment does result in less adverse effects, several research groups have remarked that respiratory depression and constipation are observed even in β-arrestin knockout animals and that so-called biased agonists can still produce these adverse effects, even if to a lesser degree [25,26]. The physiological role of β-arrestin is also central to receptor internalization and thus down-regulation of surface receptor density. This may be one mechanism by which opioid users may develop tolerance if the β-arrestin-mediated receptor internalization requires a higher dose to activate the remaining receptors. With the approval of oliceridine as a biased opioid receptor agonist, there have been hopes that such drugs would have less potential for dependence, constipation, and respiratory depression. Yet, similar to other non-biased opioids, oliceridine does present with a very similar profile and carries warnings about addiction, nausea and vomiting, and respiratory depression.

### 2.1. Indole Alkaloids as Opioid Receptor Modulators

A number of natural products containing indole structures have been found to interact with opioid receptors. Similar to morphine derived from Papaver somniferum, many other plants contain alkaloids that act on opioid receptors. Among them, the indole structure has proven to be a common feature that appears to confer less structural rigidity as well as higher flexibility to interact with additional amino acid residues on the opioid receptors. However, almost all indole alkaloids that act on opioid receptors also show affinity and pharmacological actions at other receptors, in particular, serotonin and dopamine receptors. This is attributed to the structural similarity with the neurotransmitter serotonin (5-hydroxytryptamine) and the principal structure of a phenethylamine. Reduced selectivity of these indole alkaloids leads to a complex pharmacology which may at times be antagonistic to one another. For example, action at opioid receptors may cause sedation, but activation of serotonin and dopamine receptors will lead to arousal. Both actions, depending on the individual and the location of the receptors in the periphery or central nervous system, may cancel each other out.

#### 2.1.1. Ibogaine

**Figure 2:**
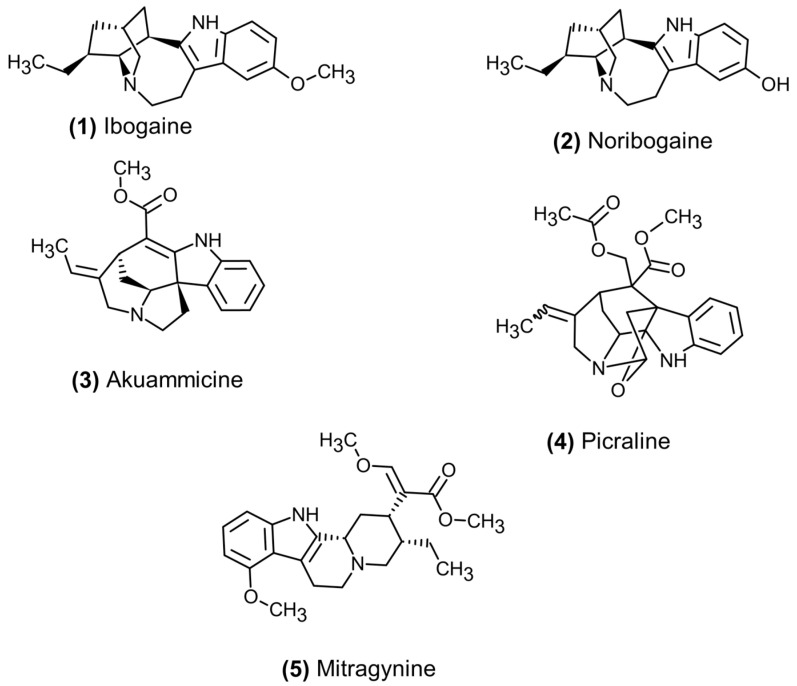
Structures of indole alkaloids with activity at opioid receptors.

Among the opioid-modulating indole alkaloids, ibogaine is isolated from the African shrub *Tabernanthe iboga* in the family of the Apocynaceae. Ibogaine and its active metabolite noribogaine act as weak agonists at μ-opioid receptors (Figure 2), as well as a range of other receptors such as serotonin, dopamine, and cholinergic receptors [27,28]. Although not investigated at the μ-opioid receptor, ibogaine and noribogaine were found to be GPCR-biased agonists at κ-opioid receptors, with only minimal recruitment of β-arrestin [29]. To date, a similar biased action has not been shown for either substance at the μ-opioid receptor (Table 1).

#### 2.1.2. Akuammicine

Another monoterpene alkaloid is akuammicine, which has been found in various Apocynaceae family plants. Most commonly among them is *Picralima nitida*, a tree native to Africa and referred to as akuamma in Ghana, the Ivory Coast, and Nigeria [30]. Similar to ibogaine, akuammicine and structurally related compounds are biased GPCR agonists that do not recruit β-arrestin [31]. Akuamma alkaloids have also been identified in various other plants, including *Vinca minor* and *Catharanthus roseus* [32,33], although they appear in trace amounts and are not likely to contribute to the pharmacological effects of those plants. In contrast, the structurally related indole alkaloids aspidocarpine, 11-methoxytubotaiwine, and picraline in *Aspidosperma cuspa* have been found to exert analgesic effects [34]. In a separate investigation, picraline and akuammicine (Figure 2) have been determined to be weak partial agonists at μ- and κ-opioid receptors and both did not recruit β-arrestin at investigated concentrations [35].

#### 2.1.3. Mitragynine

Isolated from the kratom tree, *Mitragyna speciosa* (Rubiaceae), native to Southeast Asia, is the indole alkaloid mitragynine (Figure 2), which also shows GPCR-biased activity at μ- and κ-opioid receptors as a partial agonist (Table 1) [36,37,38]. Like morphine and other classical opioids, the corynanthe-based rigid tetracyclic structure interacts with additional binding sites on opioid receptors [39,40]. The additional binding pocket that appears to be specific to indole alkaloids is with amino acid residues in transmembrane regions 2 and 3, with asparagine in position 127 and isoleucine in position 144 in addition to a tryptophan group in position 133 [41]. In addition, the methoxy group on the indole aromatic ring system appears to be necessary for the biased activation of the μ-opioid receptor through interaction with methionine in position 151 and a separate interaction with aspartate in position 147 [40].

In addition to mitragynine, its 7-hydroxyl derivative and mitragynine pseudoindoxyl presented with higher potency at the opioid receptors [40,42]. The CYP-resultant 7-hydroxymitragynine metabolite appears to have higher potency as a partial agonist at opioid receptors with a higher liability for respiratory depression relative to mitragynine. Further metabolism of 7-hydroxymitragynine in human blood results in the more potent full opioid agonist mitragynine pseudoindoxyl, indicating at least 20 times the potency of morphine with less pronounced respiratory depression as well [42,43].

**Table 1:** Naturally occurring indole alkaloids opioid receptor binding affinities, β-arrestin recruitment, and non-opioid targets. ND: not determined, NR: not reported.

Indole Alkaloid	Natural Source(s)	Opioid Receptor Binding	Maximal β-Arrestin Recruitment	Other Receptor Binding (Under 100 μM)	References
Ibogaine	*Tabernanthe iboga*	μ: 2.0 μM, κ: 2.2 μM	ND	5-HT_2A_, 5-HT_3_, D_3_, NMDA, M_1_, M_2_, N_N_	[29,44,45,46]
Noribogaine	*Tabernanthe iboga*	μ: 0.61 μM, κ: 0.62 μM, δ: 5.2 μM	13%	NMDA, M_1_, M_2_, N_N_	[29,47]
Akuammicine	*Picralima nitida*	μ: 3.31 μM, κ: 0.089 μM, δ: 23.2 μM	ND	NR	[35]
Aspidocarpine	*Aspidosperma cuspa*	NR	NR	NR	
Picraline	*Aspidosperma cuspa*	κ: 2.38 μM	ND	D_5_	[35]
Mitragynine	*Mitragyna speciosa*	μ: 0.7 μM, κ: 1.7 μM, δ: 6.8 μM	NR	5-HT_1A_, 5-HT_2A_, 5-HT_2B_, α_2A_, α_1A_, D_2_	[40,48,49]

## 3. Structural Features of Indole Alkaloids at Opioid Receptors

The indole structure is a flat two-membered aromatic ring system with a basic secondary amine. While substitution of classical opioids with an indole ring system appears to confer selectivity toward δ-opioid receptors, as is the case for naltrindole [50], the absence of the morphinan ring system, but presence of an indole-based ring, provides for several novel binding opportunities.

In a comparative binding study between mitragynine pseudoindoxyl, a metabolite of mitragynine reported in humans, and lofentanil, a highly potent fentanyl derivative, several distinct binding interactions with the μ-opioid receptor were noted [24]. Both mitragynine pseudoindoxyl and lofentanil shared a range of common amino acid interactions with the receptor, while there were distinctions that may contribute to the unique pharmacological properties observed with indole alkaloids. In particular, interactions with tyrosine 39 in transmembrane region 1, leucine 57 in transmembrane region 2, and histidine 36 in transmembrane region 7 were distinct for mitragynine pseudoindoxyl. While shared between both compounds, the glutamine residue in position 124 in transmembrane region 7 orients differently, thus potentially indicating a molecular switch that allows for biased activation of GPCR signaling in addition to the helix 8 intracellular loop. The differential orientation of the glutamine residue also allows for accommodation of the indole ring system, which may be a common mechanism for indole alkaloids at μ-opioid receptors. The orientation of the glutamine residue also may facilitate an interaction with tyrosine in position 326 that does lead to β-arrestin recruitment in lofentanil, while it does not do so for mitragynine pseudoindoxyl in this particular investigation.

The interplay of the glutamine 124 residue to accommodate the indole ring system with the lack of interaction between glutamine and tyrosine 326 in human μ-opioid receptors may thus explain the lack of β-arrestin recruitment and reduced liability for respiratory depression and other commonly observed adverse effects with classical opioids.

While this is a promising starting point for the development of novel opioid analgesics with a better side effect profile, the indole structure also confers activity at other receptor systems, which can contribute to complex pharmacological profiles. In addition, more work is needed to confirm that the different interactions are specifically leading to a biased activation of the receptor, and hence, the above should be considered as tentative hypotheses to be expanded through further in vitro binding studies and in vivo observational data indicating a clear decrease in adverse effects, particularly respiratory depression, compared to non-biased agonists.

## 4. Pharmacokinetic Parameters of Indole Alkaloids

Current understanding of the pharmacokinetics of alkaloids discussed in this review is limited. Though preclinical animal data is available, pharmacokinetic data for individual alkaloids from human clinical trials is sparse despite a long use history of the respective plant sources.

Ibogaine has been investigated for its psychoactive effects since the 1970s and is associated with a long duration of psychoactive effects (Table 2). Many of these effects, however, appear to be mediated from its metabolism to the active metabolite, noribogaine, that is generated through demethylation [47]. Ibogaine metabolism is primarily mediated by CYP 2D6 and its inhibition may lead to elevated levels of noribogaine [51]. Furthermore, poor CYP 2D6 metabolizer phenotypes may need to be given a lower dose of ibogaine to prevent adverse effects. Because ibogaine can prolong the QT interval, it is critical to obtain a comprehensive health history before using ibogaine or noribogaine in any setting [52]. Based on its cardiovascular adverse effects, ibogaine is unlikely to achieve drug approval.

Little is known about the akuamma alkaloids in regard to their pharmacokinetic properties. Both akuammicine and picraline show high permeability across the intestinal lining that is not transporter-mediated, making them feasible candidates for oral administration [57]. Akuammicine remains stable in human hepatocytes, while picraline is rapidly metabolized to inactive metabolites. To date, no in vivo pharmacokinetic data is published on either alkaloid, likely because of the low concentrations in seed extract preparations. No pharmacokinetic data on aspidocarpine has been published.

Mitragynine has been well-characterized in preclinical and clinical studies in regard to its pharmacokinetics and remains the most studied indole alkaloid from kratom. Metabolism of mitragynine to the active 7-hydroxymitragynine metabolite in the liver is mediated by CYP 3A4 [58]. The more potent 7-hydroxymitragynine is converted to mitragynine pseudoindoxyl by a plasma esterase, leading to an even more potent opioid receptor agonist [59]. Mitragynine does accumulate in the body, given its long half-life and high volume of distribution (Table 2). It shows a dose-dependent increase in volume of distribution and clearance, further supporting a distribution into deeper tissues. However, 7-hydroxymitragynine has a comparably short half-life of 3–5 h, which may explain the intermediate duration of analgesic effects [60]. Both mitragynine and 7-hydroxymitragynine inhibit the metabolic enzymes CYP 2D6 and CYP 3A, leading to potential clinically relevant drug interactions [61]. Kratom has been associated with dependence and physical withdrawal, although it appears to be milder than opioid use disorder [62]. There have also been case reports of seizures and cardiovascular events associated with consumption of high kratom doses or enriched extracts [63]. Blood concentrations of mitragynine tend to be higher in consumers who report adverse effects, although most cannot be solely attributed to kratom, but rather a polydrug exposure.

## 5. Discussion

The molecular mechanisms involved in the interaction of opioids like morphine or fentanyl relate to the observed desired effect of analgesia while also causing undesired effects including reduced gastrointestinal transit, respiratory depression, and tolerance and dependence. Opioids remain an essential therapeutic drug class to reduce and treat acute and chronic pain states and conditions that cannot be properly controlled with non-opioid medications. While there have been advances in the development of novel opioid receptor modulators, such as the approval of oliceridine in the US and other derivatives that are biased ligands at opioid receptors, several safety issues remain. In the case of oliceridine, there are concerns about QT prolongation that delayed its approval [64]. An indole derivative itself, cebranopadol is currently in phase 3 clinical trials and did not indicate substantial respiratory depression in animals and lower respiratory depression in healthy human volunteers, which has been attributed to its biased activity at opioid receptors [65]. With several G-protein biased drugs in the pipeline, it appears that analgesics with a more favorable side effect profile are likely to enter the market soon.

Ever since the initial discovery of morphine and the use of heroin, morphine, and other morphinan-based opioid receptor modulators to treat pain, there has been exploration of structurally diverse drug classes to target opioid receptors with the goal to develop drugs that relieve pain without the undesired effects commonly observed with higher opioid dose use. Further modification of the morphinan core structure led to the discovery of piperidine derivatives when fentanyl was introduced in 1968 [66]. Similarly, the benzimidazole core structure was discovered in 1957 and resulted in even more potent compounds commonly known as nitazenes [67]. Etonitazene was initially approved in the United Kingdom for clinical use but is now in schedule 1 in several countries and the United Nations [68].

However, over the last several decades, opioid misuse and abuse has led to changes in prescribing practices for opioids as a consequence of several waves of the opioid epidemic, claiming thousands of lives each year in the US alone [69]. A majority of people abusing opioids in the US were prescribed opioids for therapeutic purposes before developing tolerance and dependence that often led to obtaining illicit opioids. A commonality among opioid-involved preventable deaths is respiratory depression and arrest. While some deaths have been prevented with the availability of naloxone to reverse opioid-induced respiratory depression, more potent illicit opioids like nitazenes require aggressive rescue approaches with higher doses of naloxone that may not always be readily available [70].

As discussed in this review, naturally occurring indole alkaloids that were traditionally used for their analgesic effects do interact with opioid receptors primarily as partially biased agonists. The selective activation of the G-protein coupled to the opioid receptors leads to analgesia, while it is proposed that the absence of β-arrestin recruitment limits respiratory depression. However, none of the natural products discussed are likely to be feasible drug candidates but rather serve as a scaffold for the development of derivatives that present better pharmacokinetics and pharmacodynamics. Especially regarding off-target effects, it is notable that some of the non-opioid targets for mitragynine and ibogaine may lower the dependence liability and counteract some of the adverse effects of traditional opioid ligands. Despite this potential benefit, other adverse effects that are not desirable may arise. The indole core provides one scaffold approach that can be modified to develop a biased and opioid receptor-specific structure with improved therapeutic applications.

## Figures and Tables

**Table 2:** Non-compartmental pharmacokinetic parameters of indole alkaloids in human clinical trials. NR: not reported.

Indole Alkaloid	Half-Life (h)	Tmax (h)	Volume of Distribution (L)	Clearance (L/h)	References
Animal studies					
Ibogaine	3.3	NR	NR	5.9	[53]
Noribogaine	NR	NR	NR	NR	
Human studies					
Ibogaine	7	1	NR	0.82	[45,54]
Noribogaine	28–49	1.8–2.9	1417–3086	42.3	[45,55]
Mitragynine	43.4–67.9	1.0–1.3	3788–4855	74.7–94	[56]

## Data Availability

No new data were created or analyzed in this study. Data sharing is not applicable to this article.
